# Does timing of adjuvant chemotherapy influence the prognosis after early breast cancer? Results of the Danish Breast Cancer Cooperative Group (DBCG)

**DOI:** 10.1038/sj.bjc.6602734

**Published:** 2005-08-30

**Authors:** S Cold, M Düring, M Ewertz, A Knoop, S Møller

**Affiliations:** 1 Oncology Department R, Odense University Hospital, Sdr. Boulevard 29, DK-5000 Odense C, Denmark; 2 Danish Breast Cancer Cooperative Group, Blegdamsvej 9, 2100 København Ø, Denmark; 3 Department of Oncology, Aalborg Hospital, Aarhus University, Hobrovej 18-22, PO Box 365, DK-9100 Aalborg, Denmark

**Keywords:** breast cancer, adjuvant chemotherapy, timing, prognosis, delay

## Abstract

The purpose of this study was to examine the effect on survival of delaying the start of adjuvant chemotherapy for early breast cancer for up to 3 months after surgery. In the nation-wide clinical trials of the Danish Breast Cancer Cooperative Group, 7501 breast cancer patients received chemotherapy within 3 months of surgery between 1977 and 1999: 352 with classical cyclofosfamide, metotrexate and 5-fluorouracil (CMF); 6065 with CMF i.v. and 1084 with cyclofosfamide, epirubicin and 5-fluorouracil. For the analysis, the time between surgery and the start of chemotherapy was divided into four strata (1–3, 4, 5 and 6–13 weeks). The results show that within the three groups of chemotherapy, there was an even distribution of known prognostic factors across the four strata of initiation of chemotherapy. There was no pattern indicating a benefit from early start of chemotherapy. No significant interactions were found for subgroups of patients with a poorer prognosis (many involved lymph nodes, high-grade malignancies or hormone receptor negative disease). In conclusion, we have found no evidence for a survival benefit due to early initiation of adjuvant chemotherapy within the first 2–3 months after surgery.

It is well established that adjuvant chemotherapy reduces mortality after early breast cancer (Early Breast Cancer Trialists' Collaborative Group, 1998). The consensus panel of the St. Gallen conference has published guidelines as to which groups of patients should be offered chemotherapy and which drugs to use, but there are no recommendations on the optimal time to start chemotherapy after surgery for breast cancer (Kaufmann *et al*, 2003).

Animal models have shown circulating growth factors and accelerated growth of metastases after removal of the primary tumour (Fisher *et al*, 1989). So, from a biological point of view, early initiation would seem preferable. Perioperative chemotherapy has demonstrated some value in comparison with no chemotherapy (Nissen-Meyer *et al*, 1986), but this advantage seems to disappear when patients receive regular adjuvant chemotherapy afterwards (van der Hage *et al*, 2001).

Six studies have assessed the influence on survival of delaying chemotherapy. Two small studies from the 1980s reported an improved disease-free survival (DFS) for patients starting chemotherapy within 4 and 5 weeks compared with the patients treated later (Brooks *et al*, 1983; Pronzato *et al*, 1989). This finding was supported in a more recent Turkish study (Altundag *et al*, 2000). In a study from the MD Anderson Centre delays of 9, 10–13, 14–17 and 18+ weeks did not affect DFS (Buzdar *et al*, 1982). In a larger study of premenopausal women treated with cyclofosfamide, metotrexate and 5-fluorouracil (CMF) a DFS advantage was demonstrated only for a smaller group of patients (oestrogen receptor absent tumours) treated within 3 weeks after definitive surgery compared with the patients treated later (Colleoni *et al*, 2000). This finding could not be reproduced in a recent study from the Royal Marsden (Shannon *et al*, 2003).

As the number of patients requiring adjuvant chemotherapy is steadily increasing, often without a parallel increase in resources allocated to delivering chemotherapy, many oncology departments have to create waiting lists for starting chemotherapy. Such waiting lists cause anxiety among patients and health authorities. Since it will not be ethically acceptable to perform a trial of early *vs* late start of chemotherapy, data from other types of studies are needed to provide evidence on the effect of delays in chemotherapy on survival. Such data are available from the nation-wide clinical trial programme of the Danish Breast Cancer Cooperative Group (DBCG), which has conducted randomised clinical trials of early breast cancer since 1977.

## PATIENTS AND METHODS

### Study population

Between January 1977 and December 1999, we identified from the database of the DBCG 7690 patients with early breast cancer who received adjuvant chemotherapy. Of these, 159 (2%) patients were excluded because the initiation of chemotherapy exceeded 89 days after surgery and 30 because of missing data on tumour size, leaving 7501 patients available for analysis.

### Study design

The clinical trial programme of DBCG and the trials DBCG-77B, DBCG-82B and DBCG-82C have been described elsewhere (Andersen and Mouridsen, 1988). In the 1989 protocols, eligibility criteria for chemotherapy included node positive disease, tumours measuring more than 50 mm or malignancy grade II or III. Receptor positive, premenopausal patients were randomised to CMF i.v. *vs* castration, whereas patients with receptor-negative tumours or with unknown receptor status were randomised to CMF i.v.±pamidronate *vs* cyclofosfamide, epirubicin and 5-fluorouracil (CEF)±pamidronate. In 1998, the eligibility criteria were changed to include patients with tumours measuring more than 20 mm, and/or node positive, and/or grade II or III and/or receptor negative. In the protocols starting in 1999, pre- and perimenopausal patients were treated with CEF and if receptor positive, with tamoxifen afterwards. Postmenopausal receptor-negative patients were treated with CMF i.v. The chemotherapy regimens were classical CMF (C=100 mg m^−2^ po days 1–14, M=40 mg m^−2^ i.v. days 1 and 8 and F=600 mg m^−2^ i.v. days 1 and 8 every 4 weeks, 12 times), CMF i.v. (C=600 mg m^−2^ i.v., M=40 mg m^−2^ i.v. and F=600 mg m^−2^ i.v. every 3 weeks, 9 times) and CEF (C=600 mg m^−2^ i.v., E=60 mg m^−2^ i.v. and F=600 mg m^−2^ i.v. every 3 weeks, 9 times).

Data on prognostic factors and treatment were collected prospectively including age, tumour size, histological type, tumour grade, hormone receptor status, number of involved lymph nodes, adjuvant irradiation to chest wall and lymph nodes, adjuvant tamoxifen, and type of chemotherapy. Tumour grade was assessed according to the modified method of Elston and Ellis (1991). Hormone receptor status was determined by biochemistry and from 1990 onwards by immunohistochemistry. Tumours were classified as receptor positive by 10% or more positivity for either oestrogen or progesterone receptor or both.

The date of definitive surgery was defined as the date of the most extensive procedure ordinarily including axillary lymph node dissection, and could thus be preceded by a biopsy. Delays of chemotherapy were defined as time from definitive surgery to start of chemotherapy and categorised into the following strata: –21 days, 22–28 days, 29–35 days and 36–89 days. These strata were chosen to divide the population into four equally sized groups with one group matching the studies of Colleoni and Shannon (1–21 days).

The patients were followed by reports from the clinical departments to the DBCG at least once per year based on the civil personal registration number (a unique number assigned to all Danish residents that encodes gender and date of birth). This allows follow-up by record linkage to the Central Population Register, which keeps updated information on vital status (dates of death or emigration), and address (date of migration from the county) of all residents in Denmark. Follow-up to death is thus complete. Overall survival (OS) was defined as time from date of definitive surgery to date of death or last follow-up, which was 1 February 2003. Overall survival is reported as the best surrogate to ‘cure’.

### Statistical analysis

Baseline differences in prognostic factors between different treatment delay groups were performed by the *χ*^2^ test. Survival curves were calculated using the Kaplan–Meier method and differences assessed by the log-rank statistic. The three different chemotherapy groups were analysed separately. The Cox proportional hazards model was used to test for the independent effect of timing of chemotherapy after adjusting for known prognostic factors and hazard ratios estimated with 95% confidence limits. Tests were performed to study possible interaction between delay of chemotherapy and lymph node involvement, tumour grade and hormone receptor status.

The variables in the Cox models were as follows: *start of chemotherapy* 1–21,22–28, 29–35 and 36–89 days; *age* 45, 46–55 and 56–69 years; *tumour size*: 0–20, 21–50 and 51+ mm; *tumour type*: invasive ductal carcinoma and others; *malignancy grade*: I, II and III; *hormone receptor*: positive and negative; *involved axillary lymph nodes*: 0, 1–3, 4–6 and 7+; and *adjuvant irradiation*: ±.

## RESULTS

### Patient characteristics

**Figure 1:**
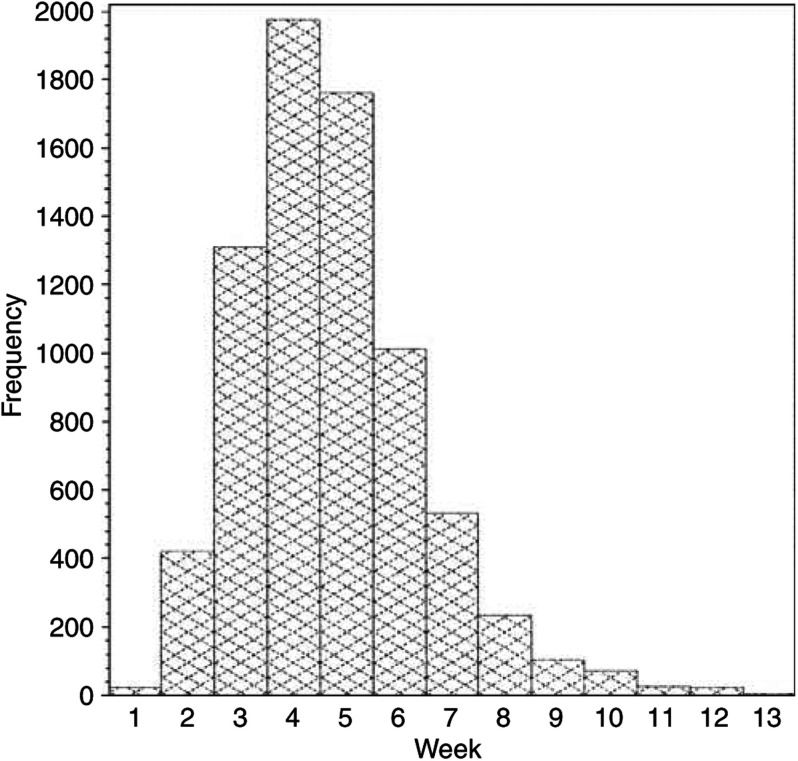
Interval from definitive surgery to start of chemotherapy in 7501 Danish patients.

A total of *7501* patients received adjuvant chemotherapy for early breast cancer within 89 days of surgery. Of these, 352 patients received classical CMF, 6065 CMF i.v. and 1084 CEF with median delays of 31, 28 and 30 days, respectively. The interval from definitive surgery to start of chemotherapy is shown in Figure 1. Approximately 25% of the patients were treated within the first 3 weeks, another 50% between week 4 and 5, respectively, whereas the remaining 25% started chemotherapy from weeks 6 to 13. According to this distribution and to match previous studies the analyses were performed in four groups of patients treated on weeks 1–3, 4, 5 and 6–13.

Tables 1, 2 and 3 show that there was an even distribution of known prognostic factors such as age, tumour size, malignancy grade, hormone receptor status and positive lymph nodes across the four strata of initiation of chemotherapy within the three groups of chemotherapy, with the exception of patients treated early with CMF i.v. being more likely to be node positive and more likely to be receptor positive than those treated later (Table 2).

### Overall survival

**Figure 2:**
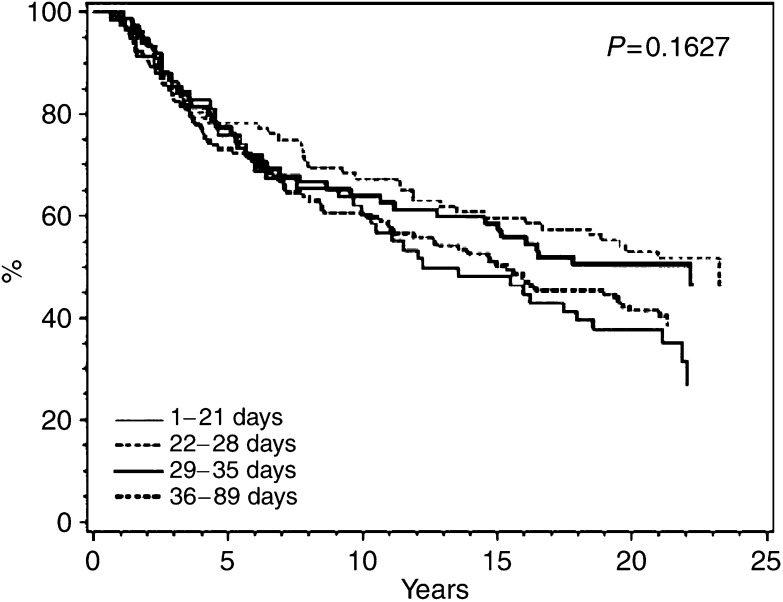
Overall survival among 352 Danish patients with early breast cancer treated with classical CMF according to interval from definitive surgery to start of adjuvant chemotherapy.

**Figure 3:**
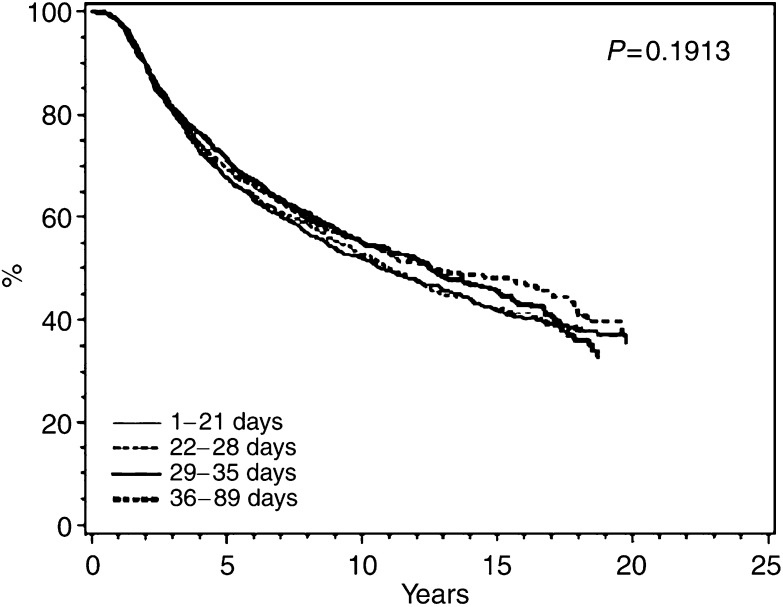
Overall survival among 6065 Danish patients with early breast cancer treated with CMF i.v. according to interval from definitive surgery to start of adjuvant chemotherapy.

**Figure 4:**
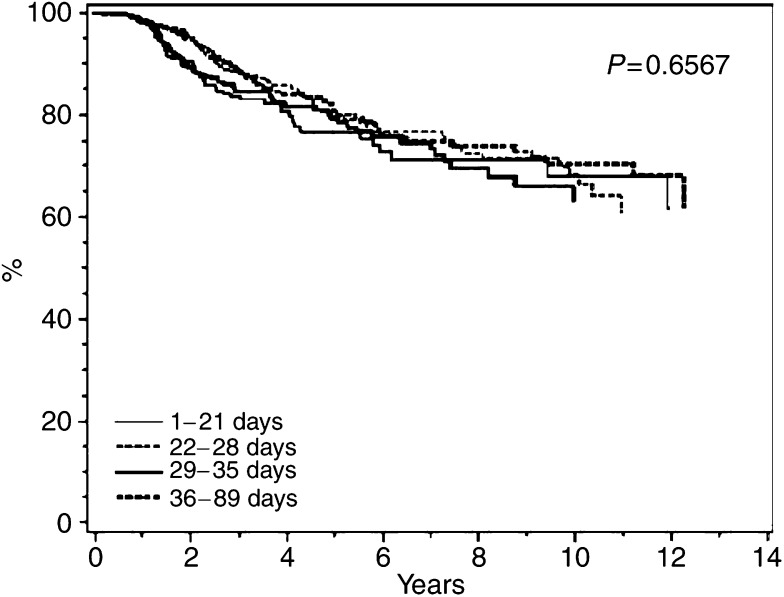
Overall survival among 1084 Danish patients with early breast cancer treated with CEF according to interval from definitive surgery to start of adjuvant chemotherapy.

Kaplan–Meier plots of OS are shown in Figures 2, 3 and 4 for classical CMF, CMF i.v., and CEF, respectively. No particular or consistent patterns were observed according to when after surgery the adjuvant chemotherapy started, the *P*-values ranging from 0.2 to 0.7. This lack of an association between start of chemotherapy and OS was confirmed in multivariate analyses taking known prognostic factors into account. Table 4 indicates a nonsignificantly increased hazard ratio for delaying start of chemotherapy for more than 4 weeks, but this estimate was based on only 352 patients receiving classical CMF. Among the 6065 patients receiving CMF i.v. (Table 5) and the 1084 patients receiving CEF (Table 6) the hazard ratios were close to unity and the associations not significant. Tests were performed for interactions between the prognostic factors shown in Tables 4, 5 and 6 and strata of initiation of chemotherapy and these did not reveal any significant interactions (data not shown).

The OS analyses have also been made according to total delay of chemotherapy (time from cytological/histological diagnosis to start of chemotherapy). However, the substitution of delay of treatment from definitive surgery by this total delay made practically no difference (data not shown). The analyses have also been performed for DFS. As the DFS curves expressed the same pattern as the OS curves, we have only reported the OS.

The proportion of patients receiving adjuvant tamoxifen was decreasing in relation to delay of chemotherapy, from 21% in the group treated within 21 days in comparison to 11% in the group treated after day 35. On the other hand, adjuvant tamoxifen did not seem to interact with chemotherapy delay and survival (data not shown).

In the three study groups 1020 patients were identified with oestrogen receptor absent tumours. For the major subgroup treated with CEF (*n*=690) delay of chemotherapy seemed of no importance (*P*=0.91).

## DISCUSSION

The present study shows that 98% of Danish breast cancer patients started adjuvant chemotherapy within 3 months of definitive surgery. Within these 3 months, we found no relation between start of chemotherapy and OS, meaning that the prognosis was similar for patients starting chemotherapy within 3 weeks after surgery to those starting chemotherapy up to 13 weeks after surgery. This is in agreement with results of other studies (Buzdar *et al*, 1982; Colleoni *et al*, 2000; Shannon *et al*, 2003). There does, however, seem to be an upper limit as to for how long time-adjuvant chemotherapy can be postponed after surgery, results from Turkey pointing towards approximately 5 months (Altundag *et al*, 2000). If chemotherapy is delayed for more than 5 months, then the concept of being adjuvant no longer holds.

The idea that an early initiation of adjuvant chemotherapy could be beneficial originates from animal models demonstrating circulating growth factors and accelerated growth of metastases after removal of the primary tumour (Fisher *et al*, 1983), and that a single dose of chemotherapy given perioperatively to mice or as an infusion within 3 days of surgery seemed more efficient than treatment given later (7 days) (van der Hage *et al*, 2001). This has also been shown in humans (Nissen-Meyer *et al*, 1986). In a meta-analysis, Clahsen *et al* (1997) found no OS advantage, whereas DFS was longer for perioperative chemotherapy than for no perioperative chemotherapy. However, this advantage was primarily found in the node negative patients who were less likely to receive further chemotherapy. When perioperative chemotherapy was combined with postoperative, adjuvant chemotherapy, perioperative chemotherapy did not improve OS (Clahsen *et al*, 1997).

Advancing the start of chemotherapy even further to preoperative or neoadjuvant treatment has also been explored. The NSABP-B18 trial demonstrated no difference in DFS in women treated with chemotherapy before surgery compared to women treated with the same chemotherapy after surgery, also indicating no benefit from early initiation of chemotherapy (Wolmark *et al*, 2001).

Two small studies have reported improved survival when chemotherapy was given early. For a subgroup of 169 patients treated with adjuvant AC, DFS was higher among patients with 1–3 involved lymph nodes treated within 4 weeks than among patients treated later, but the trend was in the opposite direction for patients with more than four involved lymph nodes (Brooks *et al*, 1983). Among 229 patients receiving adjuvant CMF i.v., OS was significantly better for the one-half of patients treated within 5 weeks in comparison with the patients treated later (Pronzato *et al*, 1989). Colleoni *et al* (2000) identified a subgroup of patients with oestrogen receptor absent tumours who had a remarkable benefit of being treated early, with 10-year DFS of 60 *vs* 34% for early *vs* late treatment, respectively. We duplicated this subgroup analysis in our material, but found no difference in OS.

The strength of the present study is that the analysis is based on a large number of patients, in particular, the group of patients treated with CMF i.v., thereby providing sufficient statistical power to detect even small differences in survival. Furthermore, the study is based on the entire Danish population for which the DBCG has issued national guidelines to ensure uniform procedures for surgery, pathology, chemotherapy, radiotherapy and follow-up. By virtue of civil personal registration numbers, there was a complete follow-up of all patients in the study.

The main limitation of this study is that it is not randomised. The initiation of chemotherapy may have been influenced by the physicians allocating the patients to ‘early’ treatment if they had a poor prognosis and the physicians did not want to delay the start of chemotherapy. In our study, patients with no involved lymph nodes were more likely to be treated late with CMF i.v. (Table 2), but on the other hand the patients treated late were more likely to be receptor negative. For the CEF-treated group the trend is similar but much less pronounced.

## CONCLUSION

In conclusion, this large population-based study did not demonstrate any benefit in OS from an early start of adjuvant chemotherapy among Danish breast cancer patients treated within 3 months of definitive surgery, or for any subgroups with potentially fast growing tumours according to increasing number of involved axillary lymph nodes, increasing malignancy grade or negative hormone receptor status. This finding is reassuring for patients who have to delay their start of chemotherapy for health reasons, for example, postoperative complications such as infections, or for other reasons.

## Figures and Tables

**Table 1:** Distribution of prognostic factors in four groups of delayed chemotherapy for patients treated with classical CMF, (percentages in brackets)

	**Patients characteristics**				
		**Start of chemotherapy in days**			
**Characteristic**	***P*-values**	**<21**	**22–28**	**29–35**	**36–89**
Total no.		58 (17)	92 (27)	75 (23)	127 (32)
*Age (years)*	0.1905				
<46		33 (17)	50 (27)	44 (23)	61 (32)
46–55		25 (16)	37 (24)	31 (20)	60 (39)
>55		0 (0)	5 (45)	0 (0)	6 (55)

*Tumour size (mm)*	0.4404				
0–20		15 (14)	29 (26)	27 (24)	40 (36)
21–50		31 (18)	39 (23)	34 (20)	65 (38)
>50		12 (18)	24 (35)	12 (18)	20 (29)
Unknown		0	0	2 (50)	2 (50)

*No. of nodes*	0.2252				
0		8 (15)	16 (29)	5 (9)	26 (47)
1–3		27 (14)	49 (26)	46 (24)	70 (36)
4–6		16 (23)	16 (23)	15 (22)	22 (32)
>6		7 (19)	11 (31)	9 (25)	9 (25)

*Histological type*	0.2185				
Ductal		54 (17)	80 (27)	61 (20)	106 (35)
Nonductal		4 (8)	11 (22)	14 (29)	20 (41)
Unknown		0	1 (50)	0	1 (50)

*Malignancy, grade*	0.4533				
I		11 (19)	13 (23)	9 (16)	24 (42)
II		35 (19)	46 (25)	40 (22)	63 (34)
III		8 (14)	20 (34)	11 (19)	19 (33)
Nonductal		4 (8)	11 (22)	14 (29)	20 (41)
Unknown		0	2 (50)	1 (25)	1 (25)

*Receptor status*	0.8585				
Negative		5 (17)	9 (31)	7 (24)	8 (28)
Positive		18 (18)	26 (27)	19 (20)	34 (35)
Unknown		35 (15)	57 (25)	49 (22)	85 (38)

*Radiotherapy*					
No		0 (0)	0 (0)	0 (0)	0 (0)
Yes		58 (16)	92 (26)	75 (21)	127 (36)

The *P*-values for the *χ*^2^-tests are calculated in the contingency tables without the unknown values.

**Table 2:** Distribution of prognostic factors in four groups of delayed chemotherapy for patients treated with CMF, (percentages in brackets)

	**Patients characteristics**				
		**Start of chemotherapy in days**			
**Characteristic**	***P*-values**	**<21**	**22–28**	**29–35**	**36–89**
Total no.	0.0435	1509 (25)	1581 (26)	1423 (23)	1552 (26)
*Age (years)*					
<46		629 (26)	641 (26)	588 (24)	574 (24)
46–55		613 (25)	617 (25)	569 (23)	648 (27)
>55		267 (23)	323 (27)	266 (22)	330 (28)

*Tumour size (mm)*	0.1083				
0–20		646 (24)	712 (26)	622 (23)	708 (26)
21–50		694 (26)	699 (26)	662 (24)	652 (24)
>50		156 (26)	155 (26)	122 (20)	170 (28)
Unknown		13 (19)	15 (22)	17 (25)	22 (33)

*No. of nodes*	<0.0001				
0		187 (16)	264 (23)	295 (26)	388 (34)
1–3		849 (27)	865 (28)	709 (23)	722 (23)
4–6		239 (26)	263 (28)	212 (23)	221 (24)
>6		234 (28)	189 (22)	205 (24)	221 (26)
Unknown		0	0	2 (100)	0

*Histological type*	0.3105				
Ductal		1301 (25)	1369 (26)	1238 (23)	1370 (26)
Nonductal		198 (27)	201 (27)	178 (24)	171 (23)
Unknown		10 (26)	11 (28)	7 (18)	11 (28)

*Malignancy, grade*	0.0651				
I		254 (26)	270 (28)	193 (20)	247 (26)
II		654 (25)	660 (25)	634 (24)	661 (25)
III		360 (23)	415 (26)	384 (24)	426 (27)
Nonductal		198 (27)	201 (27)	178 (24)	171 (23)
Unknown		43 (27)	35 (22)	34 (21)	47 (30)

*Receptor status*	<0.0001				
Negative		303 (16)	446 (24)	503 (27)	602 (32)
Positive		719 (28)	663 (26)	595 (23)	620 (24)
Unknown		487 (30)	472 (29)	325 (20)	330 (20)

*Radiotherapy*	0.9174				
No		860 (25)	912 (26)	814 (24)	876 (25)
Yes		649 (25)	669 (26)	609 (23)	676 (26)

The *P*-values for the χ^2^-tests are calculated in the contingency tables without the unknown values.

**Table 3:** Distribution of prognostic factors in four groups of delayed chemotherapy for patients treated with CEF, (percentages in brackets)

	**Patients characteristics**				
		**Start of chemotherapy in days**			
**Characteristic**	***P*-values**	**<21**	**22–28**	**29–35**	**36–89**
Total no.		188 (17)	305 (28)	263 (24)	328 (30)
*Age (years)*	0.2714				
<46		90 (18)	140 (28)	129 (26)	146 (29)
46–55		85 (19)	125 (28)	104 (23)	135 (30)
>55		13 (10)	40 (31)	30 (23)	47 (36)

*Tumour size (mm)*	0.4970				
0–20		78 (16)	148 (30)	118 (24)	156 (31)
21–50		92 (18)	137 (27)	129 (25)	149 (29)
>50		17 (25)	18 (26)	13 (19)	21 (30)
Unknown		1 (13)	2 (25)	3 (38)	2 (25)

*No. of nodes*	0.2176				
0		70 (16)	123 (28)	98 (22)	153 (34)
1–3		65 (19)	92 (27)	83 (24)	105 (30)
4–6		23 (18)	40 (31)	34 (26)	34 (26)
>6		30 (18)	50 (30)	48 (29)	36 (22)

*Histological type*	0.0009				
Ductal		164 (17)	282 (29)	250 (25)	287 (29)
Nonductal		24 (25)	22 (22)	11 (11)	41 (42)
Unknown		0	1 (33)	2 (67)	0

*Malignancy, grade*	0.0072				
I		15 (17)	22 (25)	21 (24)	29 (33)
II		68 (15)	143 (31)	112 (24)	143 (31)
III		79 (19)	115 (28)	111 (27)	110 (27)
Nonductal		24 (25)	22 (22)	11 (11)	41 (42)
Unknown		1 (11)	3 (17)	8 (44)	5 (28)

*Receptor status*	0.1634				
Negative		54 (14)	101 (27)	92 (24)	133 (35)
Positive		109 (17)	177 (29)	152 (25)	177 (29)
Unknown		25 (28)	27 (30)	19 (21)	18 (20)

*Radiotherapy*	0.4338				
No		74 (15)	138 (29)	119 (25)	153 (32)
Yes		114 (19)	167 (28)	144 (24)	175 (29)

The *P*-values for the *χ*^2^-tests are calculated in the contingency tables without the unknown values.

**Table 4:** Hazard ratios and lower and upper confidence limits (lcl–ucl) from multivariate analysis: overall survival according to prognostic factors and delay of chemotherapy among 352 Danish breast cancer patients treated with adjuvant classical CMF

**Characteristics**	***P*-values**	**Hazard ratio (lcl–ucl)**
*Start of chemotherapy*	*P*=0.2658	
1–3 weeks		1
4 weeks		0.929 (0.441–1.957)
5 weeks		1.549 (0.761–3.149)
6–13 weeks		1.588 (0.856–2.948)

*Age (years)*	*P*=0.9892	
–45		1
46–55		0.962 (0.568–1.627)
56–69		0.997 (0.285–3.480)

*Tumour size (mm)*	*P*=0.0203	
–20		1
21–50		1.843 (0.974–3.486)
51–		2.848 (1.365–5.945)

*No. of nodes involved*	*P*=0.2545	
0		1
1–3		1.783 (0.894–3.553)
4–6		1.615 (0.725–3.596)
7–		2.318 (0.978–5.498)

*Histological type*	*P*=0.9607	
Nonductal		1
Ductal		1.027 (0.355–2.975)

*Malignancy grade*	*P*=0.0553	
I		1
II		2.255 (1.007–5.052)
III		1.293 (0.457–3.656)

*Receptor status*	*P*=0.0199	
Negative		1
Positive		0.257 (0.083–0.792)

**Table 5:** Hazard ratios and lower and upper confidence limits (lcl–ucl) from multivariate analysis: overall survival according to prognostic factors and delay of chemotherapy among 6065 Danish breast cancer patients treated with adjuvant CMF i.v.

**Characteristics**	***P*-values**	**Hazard ratio (lcl–ucl)**
*Start of chemotherapy*	*P*=0.1436	
1–3 weeks		1
4 weeks		1.021 (0.903–1.155)
5 weeks		0.890 (0.782–1.012)
6–13 weeks		1.002 (0.884–1.136)

*Age (years)*	*P*<0.0001	
–45		1
46–55		0.903 (0.813–1.003)
56–69		1.346 (1.187–1.527)

*Tumour size (mm)*	*P*<0.0001	
–20		1
21–50		1.480 (1.339–1.635)
51–		2.002 (1.726–2.321)

*No. of nodes involved*	*P*<0.0001	
0		1
1–3		2.248 (1.908–2.647)
4–6		3.678 (3.072–4.404)
7–		5.621 (4.713–6.704)

*Histological type*	*P*=0.2438	
Nonductal		1
Ductal		0.896 (0.746–1.077)

*Malignancy grade*	*P*<0.0001	
I		1
II		1.537 (1.329–1.779)
III		1.775 (1.515–2.080)

*Receptor status*	*P*<0.0001	
Negative		1
Positive		0.764 (0.691–0.844)

*Adjuvant irradiation*	*P*<0.0001	
−		1
+		0.745 (0.675–0.822)

**Table 6:** Hazard ratios and lower and upper confidence limits (lcl–ucl) from multivariate analysis: overall survival according to prognostic factors and delay of chemotherapy among 1084 Danish breast cancer patients treated with adjuvant CEF

**Characteristics**	***P*-values**	**Hazard ratio** (**lcl**–**ucl**)
*Start of chemotherapy*	*P*=0.6205	
1–3 weeks		1
4 weeks		1.218 (0.800–1.854)
5 weeks		1.045 (0.716–1.525)
6–13 weeks		1.238 (0.861–1.782)

*Age (years)*	*P*=0.1709	
–45		1
46–55		0.790 (0.562–1.111)
56–69		1.090 (0.710–1.673)

*Tumour size*	*P*=0.1579	
–20		1
21–50		1.305 (0.956–1.781)
51–		1.481 (0.909–2.414)

*No. of nodes involved*	*P*<0.0001	
0		1
1–3		3.780 (2.264–6.310)
4–6		6.786 (3.928–11.723)
7–		13.297 (7.867–22.474)

*Histological type*	*P*=0.2985	
Nonductal		1
Ductal		0.676 (0.323–1.414)

*Malignancy grade*	*P*=0.0583	
I		1
II		2.064 (1.119–3.808)
III		2.080 (1.116–3.874)

*Receptor status*	*P*<0.0001	
Negative		1
Positive		0.106 (0.042–0.268)

*Adjuvant irradiation*	*P*=0.1112	
−		1
+		0.762 (0.545–1.065)
